# A purple odyssey: synthesis and structure of 3-amino-4-hy­droxy-6-oxo­cyclo­hexa-2,4-dien-1-iminium chloride monohydrate

**DOI:** 10.1107/S2056989016005107

**Published:** 2016-04-05

**Authors:** M. John Plater, William T. A. Harrison

**Affiliations:** a Department of Chemistry, University of Aberdeen, Aberdeen AB24 3UE, Scotland

**Keywords:** conjugation, anti-aromatic, hydrogen bonds, layered structure, crystal structure

## Abstract

In the cation of the title hydrated mol­ecular salt, C_6_H_7_N_2_O_2_^+^·Cl^−^·H_2_O, the six-membered ring shows unequal bond lengths consistent with delocalization of electrons over two separate 6π systems with single bonds between them. In the crystal, the components are linked by N—H⋯Cl, N—H⋯O, O—H⋯Cl and O—H⋯O hydrogen bonds, generating double layers propagating in (100).

## Abstract 2

The unequal C—C bond lengths in the six-membered ring of the C_6_H_7_N_2_O_2_^+^ cation of the title compound can be understood in terms of two separate delocalized systems.

## Chemical context   

In the course of our ongoing studies (Plater & Harrison, 2013 ▸, 2014*a* ▸,*b* ▸; Plater & Jackson, 2014 ▸) on new conjugated products obtained from the oxidation of aromatic amines, we attempted the oxidation of 1,2,4,5-tetra­amino­benzene, **1**. As long ago as 1887, it was demonstrated (Nietzki & Hagenbach, 1887 ▸) that this compound undergoes aerial oxidation to form 2,5-di­amino-1,4-benzo­quinonedi­imine, **2**. More recently, Braunstein *et al.* (2003 ▸) have studied the oxidation of compound **1** and the related compound 2,4-di­amino­resorcinol, **3**, to synthesize (1*E*)-*N*-(2,2-di­methyl­prop­yl)-5-[(2,2-di­methyl­prop­yl)amino]-2-hy­droxy-4-oxo­cyclo­hexa-2,5-dien-1-iminium chloride, **4**, which generates the zwitterion **5** when treated with base.
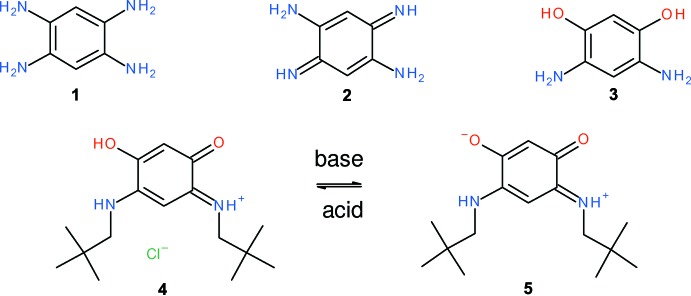


By careful oxidation of the tetra­hydro­chloride salt of amine **1** with potassium dichromate, we isolated and crystallized the chloride salt of the parent 3-amino-4-hy­droxy-6-oxo­cyclo­hexa-2,4-dien-1-iminium cation, **8**, as a monohydrate [C_6_H_7_N_2_O_2_^+^·Cl^−^·H_2_O, (I)] in the form of purple needles. This reaction must proceed *via* the elusive inter­mediate **6** which spontaneously hydrolyses. The first hydrolysis product should be inter­mediate **7**. This contains a conjugated iminium salt and a vinyl­ogous amide, which must hydrolyse rapidly, possibly because of the stability of the acidic enol formed. It appears to be a rapid hydrolysis for an amide under mild conditions and so stabilization of a tetra­hedral inter­mediate by the positive iminium salt might occur.
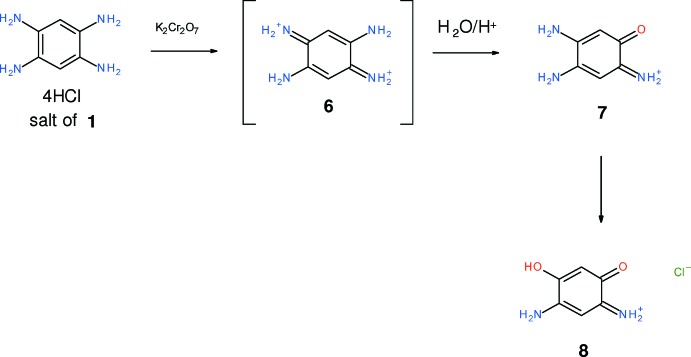


## Structural commentary   

**Figure 1:**
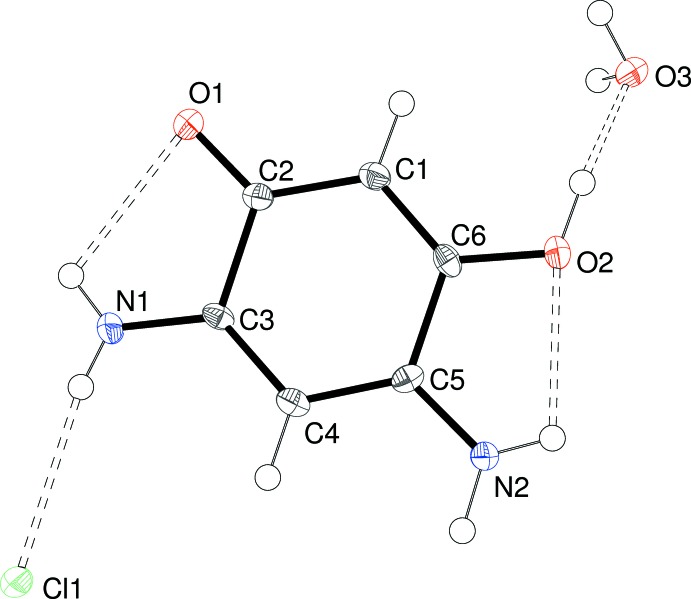
The mol­ecular structure of (I) showing 50% displacement ellipsoids. Hydrogen bonds are shown as double-dashed lines.

The asymmetric unit of (I) consists of one essentially planar C_6_H_7_N_2_O_2_^+^ cation (r.m.s. deviation for the non-hydrogen atoms = 0.028 Å), a chloride counter-ion and a water mol­ecule of crystallization (Fig. 1 ▸). Despite being a nominal 6π aromatic system, the bond lengths of the C1–C6 ring in (I) are far from equal and are split into three groups of two: the shortest are C1—C6 [1.354 (5)] and C3—C4 [1.381 (5)], followed by C4—C5 [1.406 (5)] and C1—C2 [1.436 (5) Å]. Finally, the C2—C3 [1.532 (4)] and C5—C6 [1.500 (5) Å] lengths are those expected for a C—C σ bond.
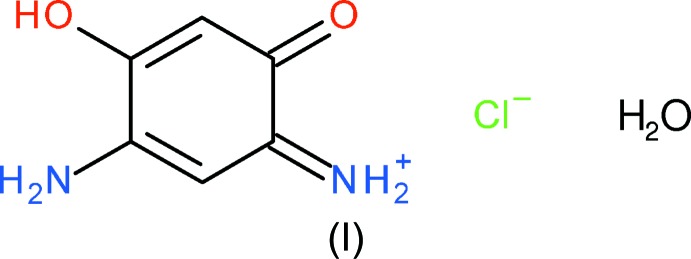


The short C3—C4 and C4—C5 bonds correlate with the approximately equal C3—N1 [1.320 (4)] and C5—N2 [1.306 (4) Å] bond lengths, which imply equal delocalization of the positive charge of the cation over atoms N1 and N2, mediated *via* the C—N and C—C bonds between them. In terms of the ‘oxygen side’ of the cation, the C6—O2 bond [1.320 (4) Å] is short for a C—O single bond whereas C2—O1 [1.227 (4) Å] is slightly lengthened for a nominal C=O double bond. This in combination with the C1—C2 and C1—C6 bond lengths again implies a degree of delocalization over these five atoms. However, the long C2—C3 and C5—C6 bonds imply little, if any, conjugation between the two delocalized components (O2/C6/C1/C2/O1 and N2/C5/C4/C3/N1) of the cation.

The cation features two intra­molecular N—H⋯O hydrogen bonds, *viz.* N1—H2*n*⋯O1 and N2—H4*n*⋯O2 (Table 1 ▸), which both close *S*(5) rings.

## Supra­molecular features   

**Figure 2:**
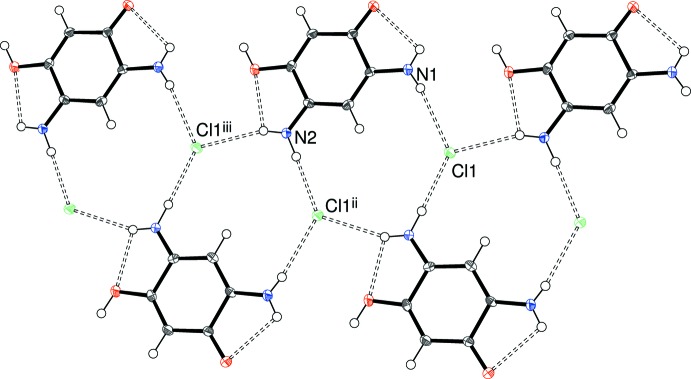
Detail of the crystal structure of (I) showing the formation of [001] chains of cations and chloride ions linked by N—H⋯Cl hydrogen bonds. Symmetry codes as in Table 1 ▸.

In the crystal, the components are linked by N—H⋯Cl, N—H⋯O, O—H⋯Cl and O—H⋯O hydrogen bonds (Table 1 ▸). If the cation and chloride anion are considered together, then [001] chains arise (Fig. 2 ▸) in which adjacent cations are related to each other by *c*-glide symmetry. Each link in the chain comprises two cations and two anions and 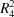
(12) loops are apparent.

**Figure 3:**
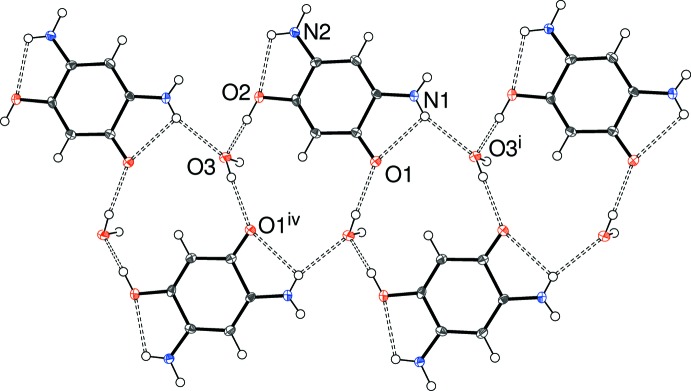
Detail of the crystal structure of (I) showing the formation of [001] chains of cations and water mol­ecules linked by O—H⋯O and N—H⋯O hydrogen bonds. Symmetry codes as in Table 1 ▸.

When the cation and water mol­ecule are considered together, an [001] chain also arises (Fig. 3 ▸). The water mol­ecule plays a key role in terms of both accepting hydrogen bonds from O2 and N1 and donating a hydrogen bond to O1 (it also acts as a donor to the chloride ion). The end result is a chain featuring 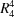
(12) loops (counted *via* the intra­molecular N1—H2*n*⋯O1 hydrogen bond).

**Figure 4:**
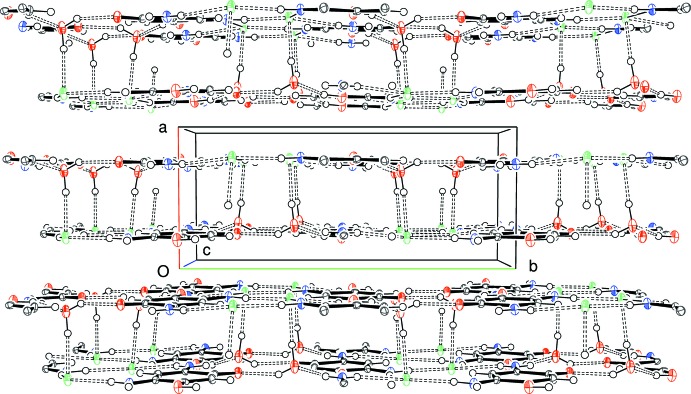
The packing in (I) viewed along [001] showing the formation of (100) double layers.

When all components are considered together, (100) double sheets result (Fig. 4 ▸), with the water-O3—H2*w*⋯Cl1 hydrogen bond providing the key link between the sheets. Overall, the chloride ion accepts four hydrogen bonds (three N—H⋯Cl and one O—H⋯Cl inter­actions) in an irregular geometry.

## Database survey   

The compound (1*E*)-*N*-(2,2-di­methyl­prop­yl)-5-[(2,2-di­methyl­prop­yl)amino]-2-hy­droxy-4-οxo­cyclo­hexa-2,5-dien-1-iminium chloride chloro­form monosolvate (CCDC refcode: VASVER; Braunstein *et al.*, 2003 ▸) was noted in the chemical context section above: these authors discuss its electronic structure in detail including its potentially anti-aromatic character. The crystal structure of the parent unprotonated zwitterion 3-oxo-4-amino-6-iminiophenolate monohydrate (HAZQUV; Yang *et al.*, 2005 ▸) is known as are those of a number of its alkyl­ated/functionalized derivatives (Braunstein *et al.*, 2009 ▸; Tamboura *et al.*, 2009 ▸; Kauf & Braunstein, 2011 ▸) and metal complexes (Paretzki *et al.*, 2010 ▸). The carbon–carbon bond lengths in the six-membered ring in all these compounds are similar to those seen in (I).

## Synthesis and crystallization   

**Figure 5:**
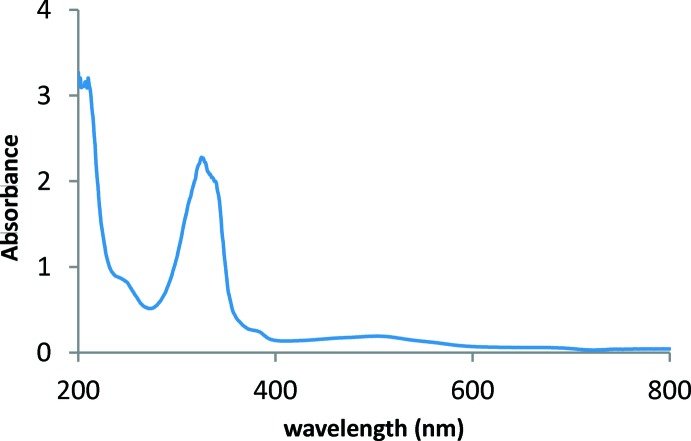
UV/visible spectrum of (I) (2.3 × 10^−4^ *M* solution in ethanol).

1,2,4,5-Benzene­tetra­amine tetra­hydro­chloride (200 mg, 0.7 mmol) in distilled water (75 ml) was treated with an excess of K_2_Cr_2_O_7_ (140 mg, 0.48 mmol, 0.6 eq) and stirred at room temperature for 24 h. The brown mixture was neutralized with NaHCO_3_ giving a brown or red precipitate, which was then extracted with CH_2_Cl_2_ (10 × 50 ml). The yellow extracts were combined, deca­nted, then stirred with methanol (50 ml) containing five drops of conc. HCl(aq). The yellow solution turned purple. This was evaporated to dryness, then the product was dissolved in methanol (50 ml) to yield a red solution and recrystallized by slow evaporation to leave the title compound (15 mg, 8%) as purple needles: m.p. > 473 K; λ_max_ (ethanol)/nm 503 (log ∊ 2.90) and 325(3.99); ν (diamond anvil)/cm^−1^ 2953*br*, 1688*s*, 1547*vs*, 1401*vs*, 1310*vs*, 1251*vs*, 1141*vs*, 871*vs*, 853*s*, 711*vs*, 654*vs*, 579*vs*, 454*s* and 420*s*; *m*/*z* (orbitrap ASAP) 139.0498 (*M* ^+^, 100%), C_6_H_7_N_2_O_2_ requires 139.0502. The UV/visible spectrum of (I) is shown in Fig. 5 ▸.

## Refinement   

Crystal data, data collection and structure refinement details are summarized in Table 2 ▸. The C-bound H atoms were geometrically placed (C—H = 0.95 Å) and refined as riding atoms. The N- and O-bound H atoms were located in difference maps and their positions were freely refined. The constraint *U*_iso_(H) = 1.2*U*_eq_(carrier) was applied in all cases. The crystal studied was found to be a twin with the components related by a 180° rotation about [001].

## Supplementary Material

Crystal structure: contains datablock(s) I. DOI: 10.1107/S2056989016005107/sj5497sup1.cif

Structure factors: contains datablock(s) I. DOI: 10.1107/S2056989016005107/sj5497Isup2.hkl

CCDC reference: 1470620

Additional supporting information:  crystallographic information; 3D view; checkCIF report

## Figures and Tables

**Table 1:** Hydrogen-bond geometry (Å, °)

*D*—H⋯*A*	*D*—H	H⋯*A*	*D*⋯*A*	*D*—H⋯*A*
N1—H2*n*⋯O1	0.81 (5)	2.33 (5)	2.653 (4)	105 (4)
N2—H4*n*⋯O2	0.84 (4)	2.23 (5)	2.595 (4)	107 (4)
N1—H1*n*⋯Cl1	0.80 (5)	2.45 (5)	3.238 (3)	169 (4)
N1—H2*n*⋯O3^i^	0.81 (5)	2.25 (5)	3.011 (4)	156 (4)
N2—H3*n*⋯Cl1^ii^	0.93 (4)	2.22 (4)	3.149 (3)	177 (4)
N2—H4*n*⋯Cl1^iii^	0.83 (5)	2.44 (5)	3.231 (3)	158 (4)
O2—H1*o*⋯O3	0.92 (5)	1.65 (5)	2.548 (4)	165 (4)
O3—H1*w*⋯O1^iv^	0.88 (5)	1.98 (5)	2.801 (4)	154 (4)
O3—H2*w*⋯Cl1^v^	1.00 (5)	2.11 (5)	3.116 (3)	176 (4)

Symmetry codes: (i) 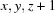; (ii) 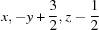; (iii) 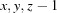; (iv) 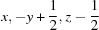; (v) 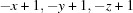.

**Table 2:** Experimental details

Crystal data	
Chemical formula	C_6_H_7_N_2_O_2_·Cl·H_2_O
*M*_r_	192.60
Crystal system, space group	Monoclinic, *P*2_1_/*c*
Temperature (K)	100
*a*, *b*, *c* (Å)	6.3070 (7), 14.9614 (18), 8.9198 (11)
β (°)	93.457 (1)
*V* (Å^3^)	840.15 (17)
*Z*	4
Radiation type	Mo *K*α
μ (mm^−1^)	0.42
Crystal size (mm)	0.11 × 0.04 × 0.03

Data collection	
Diffractometer	Rigaku Mercury CCD
Absorption correction	–
No. of measured, independent and observed [*I* > 2σ(*I*)] reflections	3102, 3102, 2789
*R*_int_	?
(sin θ/λ)_max_ (Å^−1^)	0.651

Refinement	
*R*[*F* ^2^ > 2σ(*F* ^2^)], *wR*(*F* ^2^), *S*	0.072, 0.159, 1.22
No. of reflections	3102
No. of parameters	131
H-atom treatment	H atoms treated by a mixture of independent and constrained refinement
Δρ_max_, Δρ_min_ (e Å^−3^)	0.57, −0.40

Computer programs: *CrysAlis PRO* (Rigaku, 2015 ▸), *SHELXS97* and *SHELXL97* (Sheldrick, 2008 ▸), *ORTEP-3* (Farrugia, 2012 ▸) and *publCIF* (Westrip, 2010 ▸).

## supplementary crystallographic information

### Crystal data

**Table:**

C_6_H_7_N_2_O_2_·Cl·H_2_O	*F*(000) = 400
*M**_r_* = 192.60	*D*_x_ = 1.523 Mg m^−^^3^
Monoclinic, *P*2_1_/*c*	Mo *K*α radiation, λ = 0.71073 Å
*a* = 6.3070 (7) Å	Cell parameters from 1747 reflections
*b* = 14.9614 (18) Å	θ = 3.2–27.5°
*c* = 8.9198 (11) Å	µ = 0.42 mm^−^^1^
β = 93.457 (1)°	*T* = 100 K
*V* = 840.15 (17) Å^3^	Rod, purple
*Z* = 4	0.11 × 0.04 × 0.03 mm

### Data collection

**Table:**

Rigaku Mercury CCD diffractometer	θ_max_ = 27.6°, θ_min_ = 2.7°
ω scans	*h* = −8→8
3102 measured reflections	*k* = −19→19
3102 independent reflections	*l* = −7→11
2789 reflections with *I* > 2σ(*I*)	

### Refinement

**Table:**

Refinement on *F*^2^	Primary atom site location: structure-invariant direct methods
Least-squares matrix: full	Hydrogen site location: difference Fourier map
*R*[*F*^2^ > 2σ(*F*^2^)] = 0.072	H atoms treated by a mixture of independent and constrained refinement
*wR*(*F*^2^) = 0.159	*w* = 1/[σ^2^(*F*_o_^2^) + (0.0472*P*)^2^ + 1.7994*P*] where *P* = (*F*_o_^2^ + 2*F*_c_^2^)/3
*S* = 1.22	(Δ/σ)_max_ < 0.001
3102 reflections	Δρ_max_ = 0.56 e Å^−^^3^
131 parameters	Δρ_min_ = −0.39 e Å^−^^3^
0 restraints	

### Special details

**Table:**

Geometry. All esds (except the esd in the dihedral angle between two l.s. planes) are estimated using the full covariance matrix. The cell esds are taken into account individually in the estimation of esds in distances, angles and torsion angles; correlations between esds in cell parameters are only used when they are defined by crystal symmetry. An approximate (isotropic) treatment of cell esds is used for estimating esds involving l.s. planes.
Refinement. Refined as a 2-component twin (180° rotation about [001])

### Fractional atomic coordinates and isotropic or equivalent isotropic displacement parameters (Å^2^)

**Table:**

	*x*	*y*	*z*	*U*_iso_*/*U*_eq_	
C1	0.2403 (6)	0.3972 (2)	0.3386 (4)	0.0152 (7)	
H1	0.2413	0.3419	0.2865	0.018*	
C2	0.2473 (6)	0.3979 (2)	0.4998 (4)	0.0132 (7)	
C3	0.2536 (6)	0.4888 (2)	0.5792 (4)	0.0120 (7)	
C4	0.2459 (6)	0.5671 (2)	0.4970 (4)	0.0155 (7)	
H4	0.2493	0.6233	0.5468	0.019*	
C5	0.2332 (6)	0.5632 (2)	0.3392 (4)	0.0133 (7)	
C6	0.2324 (6)	0.4747 (2)	0.2601 (4)	0.0133 (7)	
N1	0.2648 (6)	0.4838 (2)	0.7271 (3)	0.0165 (7)	
H1n	0.265 (7)	0.527 (3)	0.779 (5)	0.020*	
H2n	0.270 (7)	0.436 (3)	0.770 (5)	0.020*	
N2	0.2212 (6)	0.6344 (2)	0.2543 (4)	0.0162 (7)	
H3n	0.227 (7)	0.690 (3)	0.300 (5)	0.019*	
H4n	0.216 (7)	0.629 (3)	0.161 (5)	0.019*	
O1	0.2459 (4)	0.33037 (17)	0.5778 (3)	0.0184 (6)	
O2	0.2219 (5)	0.48297 (18)	0.1125 (3)	0.0201 (6)	
H1o	0.230 (7)	0.430 (3)	0.063 (5)	0.024*	
Cl1	0.22638 (16)	0.67344 (6)	0.89828 (10)	0.0208 (3)	
O3	0.3077 (5)	0.34226 (17)	−0.0329 (3)	0.0223 (6)	
H1w	0.247 (8)	0.294 (3)	−0.001 (5)	0.027*	
H2w	0.458 (8)	0.334 (3)	0.008 (6)	0.027*	

### Atomic displacement parameters (Å^2^)

**Table:**

	*U*^11^	*U*^22^	*U*^33^	*U*^12^	*U*^13^	*U*^23^
C1	0.019 (2)	0.0121 (15)	0.0149 (17)	0.0016 (14)	0.0001 (14)	−0.0023 (13)
C2	0.0134 (18)	0.0119 (15)	0.0146 (17)	−0.0007 (14)	0.0013 (14)	−0.0015 (13)
C3	0.0101 (17)	0.0119 (15)	0.0137 (16)	0.0012 (13)	−0.0030 (13)	−0.0036 (12)
C4	0.0170 (19)	0.0134 (16)	0.0161 (17)	−0.0001 (15)	0.0001 (14)	−0.0038 (13)
C5	0.0118 (18)	0.0113 (15)	0.0168 (17)	−0.0006 (14)	0.0005 (14)	0.0021 (13)
C6	0.0105 (18)	0.0179 (16)	0.0112 (16)	0.0006 (14)	−0.0014 (13)	−0.0031 (13)
N1	0.0243 (19)	0.0150 (14)	0.0101 (15)	0.0015 (14)	−0.0005 (13)	−0.0001 (12)
N2	0.0246 (19)	0.0122 (13)	0.0120 (15)	−0.0015 (13)	0.0038 (13)	−0.0003 (12)
O1	0.0271 (15)	0.0137 (11)	0.0143 (12)	0.0001 (12)	0.0017 (11)	0.0015 (10)
O2	0.0335 (18)	0.0165 (13)	0.0103 (12)	0.0016 (12)	0.0008 (11)	−0.0001 (10)
Cl1	0.0343 (5)	0.0136 (4)	0.0144 (4)	−0.0010 (4)	−0.0010 (4)	0.0012 (3)
O3	0.0368 (18)	0.0133 (13)	0.0170 (14)	−0.0015 (12)	0.0034 (12)	0.0015 (10)

### Geometric parameters (Å, º)

**Table:**

C1—C6	1.354 (5)	C5—C6	1.500 (5)
C1—C2	1.436 (5)	C6—O2	1.320 (4)
C1—H1	0.9500	N1—H1n	0.80 (5)
C2—O1	1.227 (4)	N1—H2n	0.81 (5)
C2—C3	1.532 (4)	N2—H3n	0.93 (4)
C3—N1	1.320 (4)	N2—H4n	0.83 (5)
C3—C4	1.381 (5)	O2—H1o	0.92 (5)
C4—C5	1.406 (5)	O3—H1w	0.88 (5)
C4—H4	0.9500	O3—H2w	1.00 (5)
C5—N2	1.306 (4)		
			
C6—C1—C2	120.6 (3)	N2—C5—C6	116.6 (3)
C6—C1—H1	119.7	C4—C5—C6	120.4 (3)
C2—C1—H1	119.7	O2—C6—C1	126.4 (3)
O1—C2—C1	124.1 (3)	O2—C6—C5	112.6 (3)
O1—C2—C3	118.0 (3)	C1—C6—C5	120.9 (3)
C1—C2—C3	117.9 (3)	C3—N1—H1n	122 (3)
N1—C3—C4	125.2 (3)	C3—N1—H2n	121 (3)
N1—C3—C2	114.2 (3)	H1n—N1—H2n	117 (4)
C4—C3—C2	120.6 (3)	C5—N2—H3n	119 (3)
C3—C4—C5	119.6 (3)	C5—N2—H4n	120 (3)
C3—C4—H4	120.2	H3n—N2—H4n	121 (4)
C5—C4—H4	120.2	C6—O2—H1o	114 (3)
N2—C5—C4	123.0 (3)	H1w—O3—H2w	102 (4)
			
C6—C1—C2—O1	176.9 (4)	C3—C4—C5—N2	178.6 (4)
C6—C1—C2—C3	−2.2 (5)	C3—C4—C5—C6	−1.3 (6)
O1—C2—C3—N1	2.4 (5)	C2—C1—C6—O2	−178.8 (4)
C1—C2—C3—N1	−178.5 (3)	C2—C1—C6—C5	0.7 (6)
O1—C2—C3—C4	−177.1 (4)	N2—C5—C6—O2	0.8 (5)
C1—C2—C3—C4	2.0 (5)	C4—C5—C6—O2	−179.3 (3)
N1—C3—C4—C5	−179.7 (4)	N2—C5—C6—C1	−178.8 (4)
C2—C3—C4—C5	−0.3 (5)	C4—C5—C6—C1	1.1 (6)

### Hydrogen-bond geometry (Å, º)

**Table:**

*D*—H···*A*	*D*—H	H···*A*	*D*···*A*	*D*—H···*A*
N1—H2*n*···O1	0.81 (5)	2.33 (5)	2.653 (4)	105 (4)
N2—H4*n*···O2	0.84 (4)	2.23 (5)	2.595 (4)	107 (4)
N1—H1*n*···Cl1	0.80 (5)	2.45 (5)	3.238 (3)	169 (4)
N1—H2*n*···O3^i^	0.81 (5)	2.25 (5)	3.011 (4)	156 (4)
N2—H3*n*···Cl1^ii^	0.93 (4)	2.22 (4)	3.149 (3)	177 (4)
N2—H4*n*···Cl1^iii^	0.83 (5)	2.44 (5)	3.231 (3)	158 (4)
O2—H1*o*···O3	0.92 (5)	1.65 (5)	2.548 (4)	165 (4)
O3—H1*w*···O1^iv^	0.88 (5)	1.98 (5)	2.801 (4)	154 (4)
O3—H2*w*···Cl1^v^	1.00 (5)	2.11 (5)	3.116 (3)	176 (4)

Symmetry codes: (i) *x*, *y*, *z*+1; (ii) *x*, −*y*+3/2, *z*−1/2; (iii) *x*, *y*, *z*−1; (iv) *x*, −*y*+1/2, *z*−1/2; (v) −*x*+1, −*y*+1, −*z*+1.
